# Impact of new talent settlement policy on housing prices: Evidence from 70 large and medium-sized Chinese cities

**DOI:** 10.1371/journal.pone.0280317

**Published:** 2023-03-24

**Authors:** Liguo Zhang, Ying Li, Chih-Chun Kung, Bingcheng Wu, Cheng Zhang

**Affiliations:** 1 School of Economics, Jiangxi University of Finance and Economics, Nanchang, Jiangxi, China; 2 School of Economics, Modern Economics and Management College, Jiangxi University of Finance and Economics, Nanchang, China; 3 School of Business Administration, Jiangxi University of Finance and Economics, Zhongding International Construction Group Co., Ltd, Nanchang, China; 4 Wu Jinglian School of Economics, Changzhou University, Changzhou, China; Al Mansour University College-Baghdad-Iraq, IRAQ

## Abstract

Since 2017, Chinese cities have set off a wave of talent migration, with major cities joining the talent war and issuing new talent settlement policies that might stimulate the real estate market through the inflow and outflow of human capital. However, the effects of new talent settlement policies on housing prices have not been extensively studied. This study used a difference-in-differences model to examine the causal effects of new talent settlement policies on housing prices in China based on data from 70 large and medium-sized cities. The results showed that new talent settlement policies had positive effects on housing prices, and the effects revealed pronounced regional heterogeneity: they were more significant in the eastern region, first-tier, and new first-tier cities, and varied across major migration zones. Further, the varying policy tools in the new talent settlement policies had disparate effects on housing prices. Thus, we recommend that new talent settlement policies must be coordinated with the goals of real estate regulation and reasonable regional standards, and that the policy tools should be tailored according to the actual conditions of cities.

## Introduction

According to China’s seventh population census results, the country experienced the largest migration in human history over the past decade [1]. In recent years, inter-city population migration in China has drawn more public attention relative to traditional urban-rural population migration [1, 2]. Inter-city population migration refers to the transfers of population from lower-tier to higher-tier cities. Since 2017, Chinese cities have taken positive action to implement new household registration policies to compete for talent. As a pioneer of this talent war, Wuhan launched the new talent settlement policy (NTSP) in 2017 [2]. Since then, almost all large- and medium-sized Chinese cities have joined this talent war. Human capital has long been viewed as the driving force of economic growth, where talents can be viewed as workers endowed with human capital [3]. High-quality city development requires a more skilled workforce, specifically in high-tech industries and tertiary sectors [4]. Hence, these targeted brought-in talents are often young, well-educated, highly trained, and attracted mainly through internal migration amongst regions [5]. Incentives play a vital role in this process. Local governments, as direct sponsors of NTSP, introduced valuable incentives to attract high-skilled migrants to boost their human capital and compete for talent. They usually formulate policy tools consisting of living subsidies, employment and entrepreneurship assistance, and social amenities. Newly arriving talents benefit directly from various policy tools, regardless of the type of company they work for. They enjoy various rewards as long as they move their hukou, the legal document recording their basic information. Policy tools and their implementation periods vary by city [1]. For example, some cities allow unconditional settlement for talent, while others offer family placement and children’s educational assistance to ’retain talent’.

Whilst the Chinese government has repeatedly emphasised that houses are for living and not speculative investments [6], this new round of national population migration renders stabilising housing prices a specific goal of the government [7]. Notably, housing prices in China have recently begun to reach unprecedented heights in major migration zones and decline in some other areas. The reason cannot be distinguished from the recent NTSP launched by local governments. NTSP increased population flow and production factors are constantly being transferred across regions. Amongst these factors, both human and financial capital can be very mobile over space. Thus, NTSP affects housing prices through population increase and disposable income growth.

**Fig 1:**
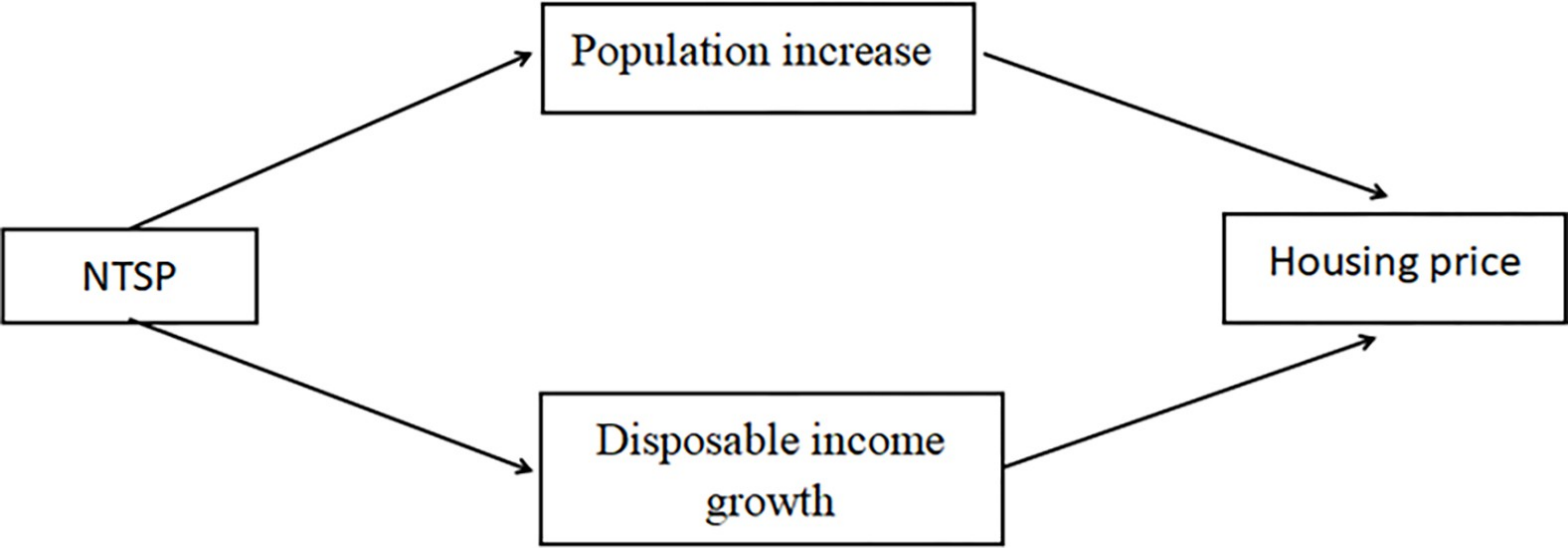
The mechanism by which NTSP affect housing prices.

The inflow of population brought about by NTSP affects the supply and demand of the real estate market, thereby inevitably pressuring housing prices. Meanwhile, policy-oriented migration will also change the expectations of prospective house buyers, so housing prices are likely to reflect the impact on housing expectations. Furthermore, the influx of talents may increase households’ disposable income. Real estate economics scholars generally believe that real estate provides housing for people and serves as a fixed asset for investment [8]; thus, capital is an essential factor that determines the price of commercial housing. Some scholars find that the inflow of people with high human capital significantly promotes regional economic development and improves local employment levels by influencing the spatial layout of enterprises [9]. A high-quality employment market significantly enhances the income level of urban residents. Housing is a normal commodity, and buyers allocate housing resources through market price competition. In China, people tend to invest in multiple houses as a means of investment allocation. Thus, an increase in income increases the pricing of commercial housing in the market from the demand side [10]. Therefore, the two key elements that can raise prices are increases in the population and in household disposable income (Fig 1).

Housing prices are closely related to people’s livelihoods and affect their quality of life and family development. This is closely related to the steady and sound development of the cities. Therefore, it is crucial to study the effects of NTSP on housing prices. However, it remains unclear whether and to what extent housing prices were affected. Additionally, the impact may vary depending on the development of cities. Therefore, it is important to explore varying impacts in different cities.

The literature on NTSP focuses mainly on the effect on upgrading urban populations and economic structures [8–10]. There is also literature from the perspective of policymakers that explores how local governments develop support policies to coordinate with NTSP [4]. In terms of the impact on housing prices, Yu [11] first proposed that NTSP may have affected housing prices, but the sample period was too early, thus making the impact insignificant at that time. Some scholars proposed a possible relationship between NTSP and housing prices, though they concentrated mainly on first-tier cities [12]. Thus, few studies focus on the relationship between NTSP and housing prices, and comparisons of different areas.

This study fills these gaps in the literature both empirically and theoretically. First, we conduct an empirical analysis using a difference-in-differences (DID) model to analyse the impact of NTSP on housing prices. We employ the instrumental variable method to address potential endogeneity and propose a new method to deal with the heterogeneity of China’s top three major migration zone. Second, in terms of theoretical contributions, we examine the impact of NTSP on the spatial difference in local house prices. Additionally, we compare the different policy tools and their corresponding effects.

We examine the causal effects of NTSP on housing prices in China using a quasi-experimental approach and a DID model based on data from 70 large and medium-sized cities collected from local government websites, the National Bureau of Statistics, and the Urban Statistical Yearbook. This study addresses the following issues:

- the extent to which NTSP affect housing prices
- heterogeneity in the policy effects among different regions and major migration zones, and
- heterogeneity in the impact of various policy tools on the implementation of NTSP.

Significantly, we find positive effects and regional heterogeneity of NTSP on housing prices in the sample period and distinctions amongst the policy tools. The results provide a reference for demographic policies in real estate markets based on the new round of population migration in China and the formulation of talent introduction policies. Additionally, it is important to study the relationship between population policies and real estate markets.

## Empirical framework

### Data description

The data in this study mainly comprised the housing price data of prefecture-level cities and the webpage data of local governments. As these policies applied mainly to large- and medium-sized cities in China, we set 2017 as the year for the first policy implementation. Therefore, the sample period was 2013 to 2019 and covered 75 large- and medium-sized cities defined by the National Bureau of Statistics in 2005, based on their economic strength, residential transaction volume, city size, and regional spread. We obtained the urban housing prices and related economic data from the China City Statistical Yearbook and China Regional Economic Statistical Yearbook. We gathered the NTSP information for all prefecture-level cities manually from the websites that host the local government reports.

### Model

To explore the effects of the NTSP on housing prices, we followed previous studies [10, 13] and constructed a housing hedonic pricing model (Model 1). We employed a quasi-experimental approach with the following DID model:

$${Lnhou\_price}_{it}=\alpha_{0}+\beta_{1}{{treat}_{t}*post}_{i}+\beta_{2}{Controls}_{it}+\gamma_{i}+\delta_{t}+\mu_{it},$$ (1)

where Lnhou_price_it_ is the outcome variable, the housing price in city i at time t, which we evaluated using the natural logarithm of the average housing price in a specific year. The key variable is the interaction between the NTSP dummy treatment and the treatment period, which is denoted by FT. FT equals one if the city implemented the policy in that year and zero otherwise. β1 and β2 are the parameters estimated in the econometric model, where β1, our variable of interest, indicates the impact of NTSP on housing prices. Controls_it_ represents the control variables, including the main economic factors affecting housing prices. After consulting the relevant literature [10], we introduced five city-level economic control variables, which included per-capita GDP (Ingdp), education expenditure (hum inv), fixed investment in real estate (hou inv), the loan balance of financial institutions (fin), and city size (citysize). To accurately estimate the impact of NTSP, we considered the different NTSP implementation times in different cities and controlled for the region fixed effect (FE) *γ*_*i*_ and time fixed effect *δ*_*t*_ [11, 14, 15]. Finally, *μ*_*it*_ is the random disturbance term, as shown in Table 1.

**Table 1:** Explanatory variables and definitions.

Variable	Variable name	Symbol	Definition
**Explained Variable**	Housing price	Lnhou_price	Natural logarithm of the price of prefecture-level commercial housing per square metre in the observed year
	Per-capita GDP	Lngdp	The logarithm of the city’s GDP in the observed year
**Control Variables**	Loan balance of financial institutions	fin	Loan balance of financial institutions in the observed year
	Investment in real estate development	hou inv	Investment in real estate development in the last year
	Education accounts for local fiscal expenditure	hum inv	The amount of education expenditure
	City size	citysize	Population density and road density

Table 2 presents the descriptive statistics of the variables used in this study. The maximum value of the housing price is 11.68, with a mean of 9.021, thus indicating heterogeneous housing prices in different cities. The average number of NTSP implementations is 0.269, thereby indicating that relatively few cities implement new talent policies. In other words, each city’s housing policy to attract talent is conservative during the sample period.

**Table 2:** Descriptive statistics.

Variable	Mean	p50	SD	Min	Max
**Lnhou_price**	9.021	8.885	0.582	8.092	11.68
**FT**	0.269	0	0.444	0	1
**Lngdp**	8.302	8.257	0.870	6.600	10.55
**hou inv**	15.49	15.36	1.021	13.01	17.94
**Fin**	2.128	2.002	1.049	0.248	6.931
**hum inv**	13.74	13.77	0.823	11.84	16.40
**Citysize**	859.0	540.5	1300	99.62	8600

### Empirical results

#### Baseline results

We employed regression analyses to examine the impact of NTSP on housing prices, taking housing prices as the outcome variable and NTSP implementation as the explanatory variable. Columns 1–3 of Table 3 report the results, including only the core explanatory variables, the city-level control variables, and controls for city and time fixed effects, respectively. Overall, the coefficients of the core explanatory variables are significantly positive. As the core explanatory variable in this study is whether the city implemented NTSP, we can better evaluate the net effects by adding time and city fixed effects. Hence, we selected the third column as the baseline regression estimation.

**Table 3:** The impact of NTSP on housing prices.

	(1)	(2)	(3)
Variable	Lnhou_price	Lnhou_price	Lnhou_price
**FT**	0.6363***	0.4700***	0.2300***
	(11.0666)	(10.3787)	(7.9139)
**Lngdp**		0.0718	0.1907**
		(0.7661)	(2.5617)
**hou inv**		0.3282***	0.0853*
		(4.0926)	(1.7937)
**Fin**		0.0368	0.0420**
		(1.5666)	(2.2435)
**hum inv**		-0.0503	-0.0140
		(-0.3914)	(-0.5181)
**Citysize**		0.0001	0.0002***
		(1.6229)	(3.8063)
**Constant**	8.8501***	3.7351***	5.9928***
	(160.7394)	(3.3852)	(7.0738)
**Observations**	490	475	475
**R-squared**	0.236	0.695	0.956
**City FE**	No	No	Yes
**Year FE**	No	No	Yes

Specifically, the regression coefficient of whether the city implemented NTSP is 0.23 and significant at the 1% level, thus indicating that NTSP can significantly enhance housing prices. It is because the NTSP relaxed the housing purchase conditions. Whilst NTSP attracted more people to the cities, thus increasing the demand for housing purchases and increasing housing prices, the favourable policies and incentives of the NTSP hasretained local talent, thus reducing the outflow of local talent and increasing housing prices.

Regarding the control variables, the regression coefficient of the region’s economic development is 0.1907 and significant at the 5% level. Every 1% increase in per-capita GDP will increase housing prices by 0.1907%. It is because economic development improves with the growth in residents’ income, thus stimulating their consumption and improving their purchasing power. Therefore, it affects the increase in housing prices. The regression coefficient of real estate investment is 0.0853 and significant at the 5% level, thereby indicating that every 1% increase in real estate investment will increase housing prices by 0.0853%. Housing is both a consumer good and a speculative asset. Real estate and the increasing demand for housing lead to an insufficient supply of housing overall, which result in a rise in housing prices. The regression coefficient of financial development is 0.0420 and significant at the 5% level, thus indicating that the housing price will rise by 0.0420% when regional financial development increases by 1%. It is because improvements in financial development will increase residents’ consumption credit, thus stimulating purchasing power and placing upward pressure on housing prices. The regression coefficient of educational expenditure is not significant, which indicates that educational expenditure has no impact on housing prices. The regression coefficient of city size is 0.0420 and significant at the 1% level, thus suggesting that housing prices will rise by 0.0002% when city size increases by 1%.

### Robustness test

We conducted several robustness checks to improve the reliability of the estimation results.

#### Parallel-trend test

In the DID model, the impact of the event itself must be exogenous to evaluate the economic effect of the event accurately. In our context, if there were differences in housing prices between cities with and without NTSP before any cities adopted NTSP, the different development paths of the cities themselves explain our results. In other words, if there was no significant difference in housing prices between the cities that did and did not implement NTSP, then the housing prices in these cities increased significantly faster after the policy implementation; that is, NTSP influenced the results.

**Fig 2:**
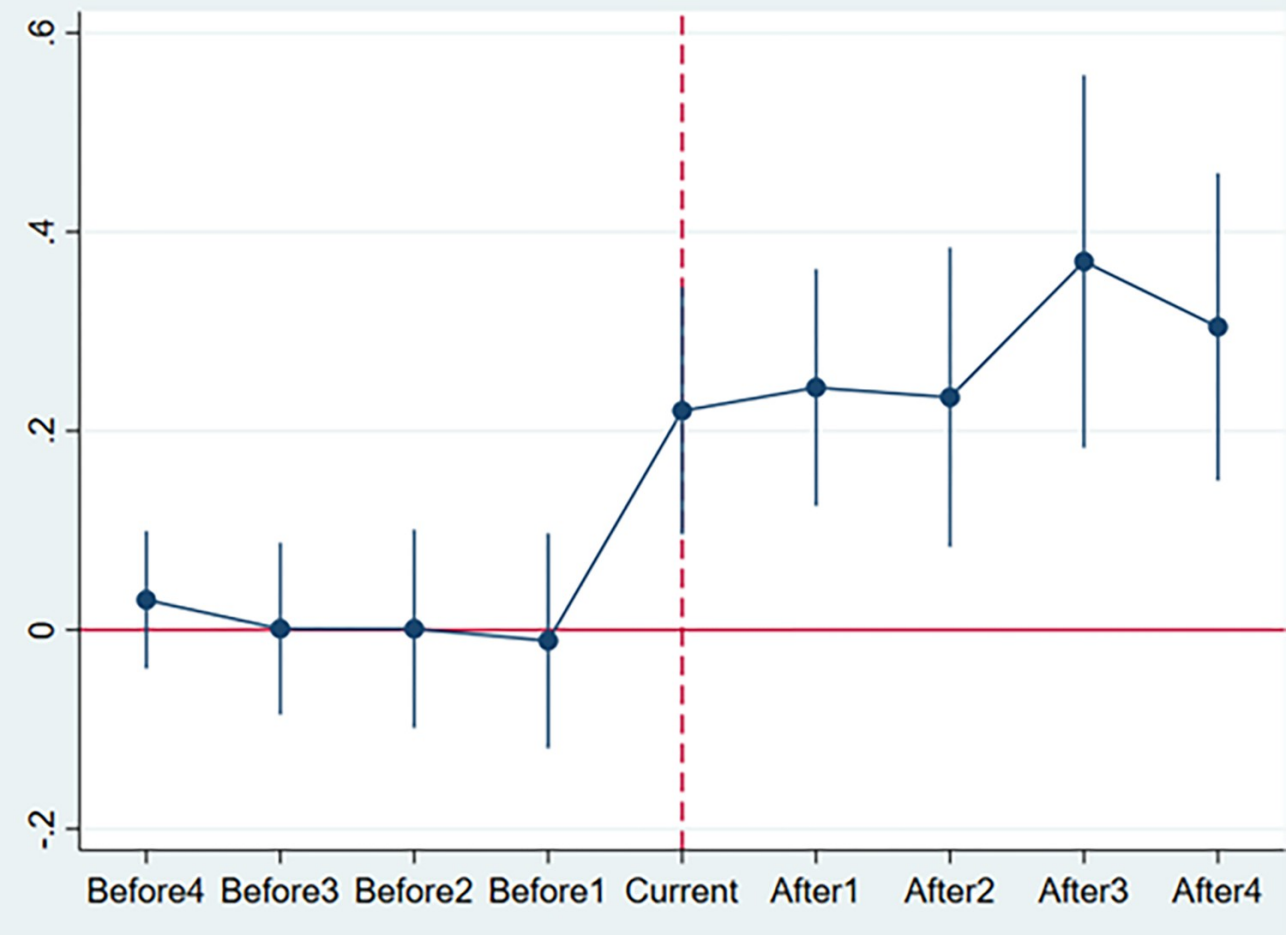
Parallel-trend test.

Fig 2 illustrates the parallel-trend test results. We see that before the implementation of NTSP, the regression coefficients of the before4–before1 period are not significant, thus indicating no significant differences in housing prices. After the implementation of NTSP, the regression coefficients of the current–after2 period are all significantly positive, which indicate that housing prices increased significantly in the year of implementation and the three years thereafter, whilst the regression coefficients of after3 and after4 are not significant, thereby denoting that the NTSP implementation would not lead to indefinite housing price increases.

#### Placebo test

We advanced the launch time of the policy by one and two years; the corresponding regression estimation results are shown in Table 4. The regression coefficients are not significant, thus indicating that the NTSP implementation causes an increase in housing prices.

**Table 4:** Placebo test.

Variable	(1)	(2)
	Lnhou_price	Lnhou_price
**F.FT**	-0.0073	
	(-0.2804)	
**F2.FT**		-0.0060
		(-0.2276)
**Lngdp**	0.1494	0.1557
	(1.1939)	(1.2310)
**hou inv**	0.1276**	0.1257**
	(2.3134)	(2.2725)
**Fin**	0.0669***	0.0680**
	(2.9506)	(2.9728)
**hum inv**	-0.0110	-0.0107
	(-0.3348)	(-0.3244)
**Citysize**	-0.0003***	-0.0003**
	(5.1436)	(5.1063)
**Constant**	5.5268***	5.4973***
	(6.1311)	(6.0431)
**Observations**	408	403
**R-squared**	0.946	0.945
**City FE**	Yes	Yes
**Year FE**	Yes	Yes

#### Sample excluding mega cities

To avoid interference from mega cities in the estimation results, we removed four mega cities: Beijing, Shanghai, Guangzhou, and Shenzhen. The corresponding estimation results in Table 5 demonstrate that after deleting these samples, the coefficient sign of the core explanatory variables is still significantly positive, thereby indicating that the estimation results in this study are reliable.

**Table 5:** Sample excluding mega cities.

	(1)	(2)	(3)
Variable	Lnhou_price	Lnhou_price	Lnhou_price
**FT**	0.6111***	0.5029***	0.2245***
	(16.6751)	(12.1953)	(7.8569)
**Lngdp**		0.0431	0.2123***
		(0.4689)	(3.1051)
**hou inv**		0.3466***	0.0428
		(4.7477)	(1.0462)
**fin**		0.0210	0.0386**
		(0.8966)	(2.2424)
**hum inv**		-0.1442	-0.0172
		(-1.1917)	(-0.7147)
**citysize**		0.0000	-0.0001
		(0.7552)	(-1.2904)
**Constant**	8.7811***	5.0261***	6.7401***
	(201.3417)	(5.0166)	(9.8475)
**Observations**	462	447	447
**R-squared**	0.309	0.652	0.942
**City FE**	No	No	Yes
**Year FE**	No	No	Yes

### Endogeneity

#### Propensity score matching (PSM) regression

As regions with higher housing prices usually have a higher level of economic development, which makes it easier to implement NTSP and attract more talent, this factor may lead to endogeneity in the model, thus affecting the reliability of the estimated results. We adopted the PSM method to solve this problem and performed a 1:1 nearest-neighbour matching between policy-implemented and non-implemented cities. We conducted the regressions based on the matched samples. Table 6 presents the results, where the coefficient of the core explanatory variables remain significantly positive, thus indicating that endogeneity does not affect our estimation results.

**Table 6:** PSM regression results.

	(1)	(2)	(3)
Variable	Lnhou_price	Lnhou_price	Lnhou_price
**FT**	0.4380***	0.3967***	0.1719***
	(7.9712)	(7.8588)	(6.4340)
**Lngdp**		0.0620	0.1687***
		(0.6680)	(2.9852)
**hou inv**		0.3004***	0.0520
		(3.8625)	(1.2329)
**fin**		0.0511*	0.0337
		(1.9272)	(1.5946)
**hum inv**		-0.0290	-0.0295
		(-0.2211)	(-1.4179)
**citysize**		0.0001	-0.0002
		(1.3297)	(-1.1059)
**Constant**	8.8682***	3.9547***	7.1955***
	(155.2725)	(3.5673)	(11.7351)
**Observations**	421	421	421
**R-squared**	0.126	0.602	0.953
**City FE**	No	No	Yes
**Year FE**	No	No	Yes

#### Instrumental variable regression

As in prior studies [16], we used historical data to create instrumental variables. The history of the relevant institutions can have a long-term impact on the development of modern society directly or through the cultural characteristics it cultivates [17]. In a feudal society, an institutionalised imperial examination was the main way to achieve vertical social mobility [18]. The civilian class could achieve upward mobility through imperial examination. Over time and through intergenerational inheritance, the imperial examination formed a unique elite group that respected knowledge and talents. This cultural feature persisted for a long time after the abolition of the imperial examination system.

Shen [18] investigated the continuous impact of the imperial examination system on contemporary human capital and found a strong positive correlation between the success rate of the imperial examination during the Qing Dynasty (measured by the number of Jinshi scholars) and contemporary human capital (measured by the number of years of modern education). From this perspective, the historical influence of the imperial examination system relates to the social mobility of contemporary regions. We adopted Shen’s [18] method and employed the number of Jinshi scholars in each city during the Qing Dynasty as an instrumental variable. Considering that this variable does not change over time, as in Nunn and Qian [19], we interacted the variable with the growth rate of educational investment at the national level and used the result as the final instrumental variable. Table 7 presents the estimation results. The sign of the coefficient of the core explanatory variable is still significantly positive after the instrumental variable regression, thus indicating that our estimation results are robust after the instrumental variable analysis.

**Table 7:** Instrumental variable regression.

	(1)	(2)	(3)
Variable	Lnhou_price	Lnhou_price	Lnhou_price
**FT**	1.8229***	0.7290***	0.3897***
	(4.3670)	(4.3613)	(4.8871)
**Lngdp**		0.0672	0.2058**
		(0.7174)	(2.5343)
**hou inv**		0.3162***	0.0661
		(3.8329)	(1.3246)
**Fin**		0.0364	0.0406**
		(1.5243)	(2.0315)
**hum inv**		-0.0620	-0.0205
		(-0.5059)	(-0.8195)
**Citysize**		0.0001	0.0002***
		(1.6414)	(3.1120)
**Constant**	8.5304***	4.0517***	
	(79.1718)	(3.7480)	
**Observations**	490	475	475
**City FE**	No	No	Yes
**Year FE**	No	No	Yes
**Kleibergen-Paap rk LM statistic**	34.875***	31.704***	19.987***
**Kleibergen-Paap rk Wald F statistic**	38.805	11.629	22.193

#### Heterogeneity analysis

Given the regional differences in the local governments’ support for NTSP and the varying endowments amongst cities, the impact of NTSP on housing prices may vary across regions. Hence, we further divided the sample into eastern, central, western, and north-eastern regions for the empirical analysis according to the typical regional division method that has been adopted by China since the 1980s [14]. The division is based on the differences in natural conditions, economic resources, transportation conditions, and other aspects. Furthermore, we divided the samples into China’s three largest major migration zones because population flow often has an obvious metropolitan effect [6]; therefore, it is common for scholars to analyse the heterogeneity of these economic zones and major migration zones. Tables 6 and 7 present the corresponding regression estimation results.

In Table 8, the coefficient of the core variable is significantly positive at the 1% level only in the eastern and central regions, whereas the western and north-eastern coefficients are relatively insignificant. In particular, the eastern region is characterised by better healthcare and education. In the context of NTSP, more people prefer to migrate to eastern cities to enjoy better social programmes; in turn, the NTSP in eastern cities have the most significant impact on housing prices. Although several policies conducive to attracting talent were introduced in the central and western regions, the impact of NTSP on housing prices is still limited. It may be because city resource endowment in these regions cannot be compared with that in the eastern region. The north-eastern regions of China face an excessive supply of real estate and a net population outflow. Though the talent war in Northeast China is fierce and these areas offered many incentives, they experienced a net outflow of population and excess real estate supply in recent years, thus making real estate markets sluggish. Therefore, the NTSP have little effect in reality, which is consistent with the results of this study.

**Table 8:** Subregional regression.

	(1)	(2)	(3)	(4)
Variable	The Eastern Region	The Central Region	The Western Region	The North-eastern Region
**FT**	0.2304***	0.2321***	0.1583**	0.1837
	(4.4153)	(3.9764)	(2.3675)	(2.5705)
**Lngdp**	0.1374	0.3017**	0.7973*	0.5381***
	(1.2576)	(2.6546)	(2.0869)	(5.0335)
**hou inv**	0.0954	0.0375	0.1046	-0.1800*
	(1.4990)	(0.4582)	(0.7490)	(-2.3633)
**fin**	0.0284	0.0323	0.0742*	0.0023
	(1.5353)	(0.4248)	(2.0108)	(0.0304)
**hum inv**	-0.0124	0.0946	0.0068	0.0893
	(-0.1132)	(0.8695)	(0.2439)	(0.6766)
**citysize**	0.0002***	-0.0001	-0.0002	-0.0004**
	(5.0905)	(-0.8281)	(-0.8143)	(-3.0480)
**Constant**	6.4226***	4.4853**	0.4879	6.4338**
	(4.2064)	(2.5276)	(0.1527)	(3.1890)
**Observations**	196	143	102	51
**R-squared**	0.971	0.914	0.902	0.978
**City FE**	Yes	Yes	Yes	Yes
**Year FE**	Yes	Yes	Yes	Yes

In the urban agglomeration grouping regression analysis (Table 9), the core variable is significantly positive in the Yangtze and Pearl River Delta regions. Additionally, the promoting effect on the Yangtze River Delta region is the largest, and that on the Beijing-Tianjin-Hebei region is not significant. The results can be analysed from several aspects. First, in terms of the scale of migration, according to the 2019 data, the proportion of inter-provincial migration in the Beijing-Tianjin-Hebei region accounts for 48.18% of its total population, which is less than 50%. Migration is concentrated in counties rather than provinces, and the migrating county population already has housing properties, thus leading to relatively weak housing purchases. However, migration in the Pearl River Delta and Yangtze River Delta regions is dominated by inter-provincial flows and reaching more than 50%. These migrants are usually just-in-need buyers, who show a strong intention and ability to purchase a property.

**Table 9:** Regression classified by major migration zones.

	(1)	(2)	(3)
Variable	The Yangtze River Delta region	The Pearl River Delta region	The Beijing-Tianjin-Hebei region
**FT**	0.4218***	0.6742*	0.2079
	(8.9594)	(4.2812)	(1.3291)
**Lngdp**	2.3258***	5.4404	0.3276
	(3.7670)	(2.8513)	(1.5371)
**hou inv**	0.1102	1.1094*	0.2091
	(0.8940)	(3.8624)	(1.7384)
**fin**	0.1713*	-0.1738**	-0.0409
	(2.1636)	(-8.4852)	(-0.2003)
**hum inv**	0.0155	-0.0099	-0.0174
	(0.2959)	(-0.0166)	(0.4086)
**Constant**	-12.8597**	-58.5915	3.1766
	(-3.1356)	(-2.7970)	(1.3060)
**Observations**	56	21	35
**R-squared**	0.982	0.991	0.970
**City FE**	Yes	Yes	Yes
**Year FE**	Yes	Yes	Yes

Second, in terms of the degree of population agglomeration, the distribution of the floating population growth in the Beijing-Tianjin-Hebei region is concentrated in the central city (Beijing) with significant polarisation effects. The data reveal that 49% of the inflow population is concentrated in Beijing, where the settlement policy is very strict, as is the government’s regulation of the real estate market. Therefore, NTSP have a limited influence on housing prices. However, the inflow distribution of the population in the Pearl River Delta and Yangtze River Delta regions is relatively dispersed and even, which does not lead to excessive population agglomeration.

Third, from the perspective of population age structure, the average age of the migrating population is highest (37.16 years old from 2013 to 2019) in the Beijing-Tianjin-Hebei region, followed by the Yangtze River Delta and Pearl River Delta regions, with the strongest housing demand.

#### Further analysis

To assess the effect of different policy tools, we distinguished the policy tools in the various NTSP in detail and set dummy variables as the main explanatory variables. We found that different cities have different policy tools and implementation times. We sorted the NTSP in different cities, which directly affect the implementation effect of the policy and its impact on the real estate market. For example, some cities offer unconditional hukou settlements to ’attract talent’, while others provide one-off rental subsidies to ’retain talent’. The specific setting is as follows: **rent subsidies**, including the provision of housing rental subsidies (monthly or one-off) as stipulated in the policy. **One-off allowance** entails explicitly cancelling the housing purchase restriction policy, house purchase subsidy, preferential house purchase price, preferential house purchase loan, and so on. **Entrepreneurship support** mainly comprises types of support such as scientific research allowances, innovation and entrepreneurship subsidies, and research platform construction funds for academic workstations and post-doctoral research workstations. **Affordable housing** refers to the government’s provision of talent apartments, public rental housing, and other supporting facilities such as **unconditional settlements.** The government implements unconditional settlements and provides family placements and children’s educational assistance. Local governments also offer incentives. The criteria for these incentives are educational background, age, and other factors of talent rather than occupational category. Local employers may also provide additional incentives for talent, but we do not include these measures in in this study because they are outside the scope of NTSP.

Table 10 lists the effects of different policy tools on housing prices. We can see that rent subsidies, one-off allowances, and unconditional settlement are significant at the level of 1%. Entrepreneurship support is also relatively significant, whilst government-subsidised housing is negative. This result may be because NTSP incentives such as financial rewards, unconditional settlement, and entrepreneurship support may stimulate local real estate prices through the inflow of human capital and financial capital, thus leading to a higher purchasing intention for housing and changing the expectations of residents. Whilst offering government-subsidised housing is another case, it can theoretically be an excellent policy tool, especially as it guarantees the housing supply and ensures talents’ welfare whilst avoiding overstimulation of the real estate market. It can be a highly effective means to resolve the housing price problems brought about by NTSP.

**Table 10:** The impact of different policy tools on housing prices.

Variable	(1) Lnhou_price	(2) Lnhou_price	(3) Lnhou_price	(4) lnhou_price	(5) lnhou_price
**Rent subsidies**	0.2088***				
	(6.6805)				
**One-off allowance**		0.2624***			
		(8.4104)			
**Entrepreneurship support**			0.2118***		
			(2.4752)		
**Affordable housing**				0.0059	
			(	(-0.1631)	
**Unconditional settlement**					0.1663***
					(3.4533)
**citysize**	0.0002***	0.0003***	0.0002***	0.0002***	0.0002***
	(4.3001)	(4.8170)	(4.8380)	(4.9113)	(4.6796)
**lngdp**	0.1787**	0.1424**	0.1207	0.1682**	0.1638**
	(2.3443)	(2.1873)	(1.6455)	(2.1923)	(2.0378)
**hou_inv**	0.1264**	0.1067**	0.1213**	0.1131**	0.1110**
	(2.5014)	(2.3886)	(5.7001)	(2.1832)	(2.1130)
**fin**	0.0528**	0.0448**	0.0473**	0.0443**-	0.0384**
	(2.3483)	(2.3673)	1.9967)	(2.2499)	(2.1328)
**hum_inv**	-0.0166	-0.0163	-0.0050	-0.0048	-0.0046
	(-0.4664)	(-0.4355)	(-0.1385)	(-0.1334)	(-0.1303)
**Constant**	5.4960***	6.0395***	5.9314***	5.6613***	5.7653***
	(5.9796)	(7.1394)	(6.5690)	(6.0769)	(6.6255)
**Observations**	471	471	470	471	471
**R-squared**	0.954	0.959	0.953	0.946	0.949
**city FE**	Yes	Yes	Yes	Yes	Yes
**Year FE**	Yes	Yes	Yes	Yes	Yes

Additionally, not all policy tools were adopted in all regions. In the previous discussion, the impact of the NTSP on housing prices was characterised by apparent heterogeneity and differences in urban endowments. Therefore, when formulating policy tools, it is crucial to promote affordable housing in popular areas with significant influence, such as the eastern region and major migration zones of the Yangtze River Delta and Pearl River Delta. First, encouraging affordable housing in these popular areas rather than directly providing money will prevent local housing prices from rising due to monetary stimuli, thus restraining housing prices to some extent. Furthermore, the orderly promotion of affordable housing will relieve the pressure on housing buyers in these cities, thus attracting talent and preventing population outflow. Other cities where the NTSP does not significantly impact housing prices should be fully open to household registration and adopt various incentives to maximise the chances of retaining talent.

## Discussion

### Conclusions

Based on unbalanced panel data from 70 large and medium-sized cities, we employed a multiphase DID model to examine the causal effects of NTSP on housing prices in China and explored the impact of heterogeneity across regions and major migration zones. In addition, we compared the effects of various policy tools innovatively. The specific research conclusions are as follows. First, NTSP has positive effects on housing prices. Second, the impact of NTSP on housing prices shows evident regional heterogeneity: it is more significant in the eastern region and central cities and varies in major migration zones. Finally, different policy tools have disparate effects on housing prices; that is, in the sample period, compared with one-off allowances, rent subsidies, entrepreneurship support, and affordable housing do not stimulate housing prices. Such policy tools should be considered more in regions where NTSP has significant effects on housing prices.

Currently, real estate economics is a hot research field, and many scholars focused on purchase restriction policies. As a new policy, NTSP will be implemented for a long time, though there is little current research to date. From a theoretical perspective, this study not only enriches the comprehensive research on NTSP but also extends the research on the price drivers of real estate, thus providing a new perspective and reference for future discussions on such issues.

### Policy implications

This study concludes that NTSP will significantly increase housing prices. To attract talent, local governments should be aware of the effects of such policies on housing price regulations. Measuring the real estate bubble of cities could exclude the impact of NTSP to formulate reasonable and practical policies. Additionally, the phenomenon of talent moving from lower-tier to higher-tier cities and clustering in major migration zones will be a long-term development trend in China as the talent war becomes increasingly fierce. Therefore, local governments that join the talent war must be aware that their local housing prices will respond to their talent settlement policy. Additionally, governments controlling housing prices should actively adjust their management policies to cope with talent wars.

The empirical results of this study identify significant regional differences in the impact of NTSP on housing prices. Accordingly, the impact of NTSP on housing prices is significantly positive in the eastern and central regions, whereas it is insignificant in the northeast. For the major migration zones, NTSP had the most significant impact on the Yangtze River Delta. China’s urbanisation process will continue to deepen. The trend of inter-city population migration will show new changes, and developed regions and core cities will undertake population spillovers by virtue of their resource endowments and locational superiority as the main forces attracting migrating population. Therefore, local governments in these areas must plan to cope with the impact of NTSP on the real estate market. Areas experiencing greater impacts on housing prices are those in the eastern region, new first-tier cities, and the Yangtze River Delta. Therefore, these cities need to pay more attention to optimising spatial population distribution and realising work-housing balance. The population growth in central cities must be strictly controlled. In addition, it is necessary to optimise the layout of transportation and industries to improve the population distribution, thus easing the pressure on the real estate market due to population agglomeration in central cities. In other words, local governments participating in the talent war should formulate differentiated policies on real estate according to their geographical differences and development, and avoid a one-size-fits-all policy-making.

From the results of the analysis of specific policy tools, more attention should be paid to innovative NTSP tools. It is essential to choose policy tools conducive to the sound development of real estate and discuss the complementary effect between talent introduction and real estate policy. Whilst introducing effective policy tools to improve talent introduction efficiency, governments should consider their negative impacts on other economic factors. For example, almost all the policy tools selected in this study achieved good results in attracting talent, thereby confirming that welfare policies such as family relocation and employment security contribute crucially to the willingness of talent to relocate. However, our empirical results suggest that policymakers should advocate for affordable housing because of its practical convenience for talent and to avoid the impact of a talent influx on the housing market. However, owing to various practical problems, many local governments still choose to substitute government-subsidised housing with monetary subsidies. Local governments should explore an integrated management system for renting, selling, and supplementing talent-subsidised housing and develop a new mode that combines government management with market operations. Some cities in China, such as Shenzhen, perform well in this regard. Moreover, it is essential to take the initiative in developing NTSP according to the characteristics of the city, give full play to the internal advantages of the city itself, explore effective tools to retain talent at the institutional level, such as public resource facilitation and financial system support, and formulate appropriate policies according to urban location and development status to minimise population outflow and attract talent.

### Research limitations and prospects

This study has a few limitations. The most important scope for improvement is that as NTSP exist within long-term urban development plans, long-term tracking of the policy should be carried out to objectively and comprehensively measure its impact.

Future research can improve this study in three ways. (1) Data: owing to limited data availability, we used data on the national average housing prices from 2013 to 2019. Pending updates to the data, it is necessary to add the latest second-hand housing and rental data to further verify this study’s main conclusions. (2) Policy Tools: we mainly discussed the impact of NTSP on housing prices, whilst the discussion on diverse policy tools is shallow. Follow-up research should further divide policy tools to analyse the implementation and effects of various tools theoretically. (3) Mechanism by which NTSP affects housing prices: the sample data period is not long enough to follow the mechanism influencing population income. Therefore, this study discussed the mechanism of NTSP on housing prices only at the theoretical level. In the future, it is desirable to empirically test the mechanism using updated data.

## Supporting information

S1 Data (DTA)
